# Supplementary material for: The effect of the definition of ‘pandemic’ on quantitative assessments of infectious disease outbreak risk
Source: Sci Rep. 2021 Jan 28;11:2547. doi: 10.1038/s41598-021-81814-3 (PMC7844018; doi:10.1038/s41598-021-81814-3)
Supplement: Supplementary file 1 — Supplementary Figures. [file 41598_2021_81814_MOESM1_ESM.pdf]

The effect of the definition of ‘pandemic’ on quantitative assessments of infectious disease outbreak risk: Supplementary Information

Benjamin J Singer[1*], Robin N Thompson[2,3], and Michael B Bonsall[1]

[1]Department of Zoology, University of Oxford
[2]Christ Church, University of Oxford
[3]Mathematical Institute, University of Oxford
[*]benjamin.singer@bnc.ox.ac.uk

January 18, 2021

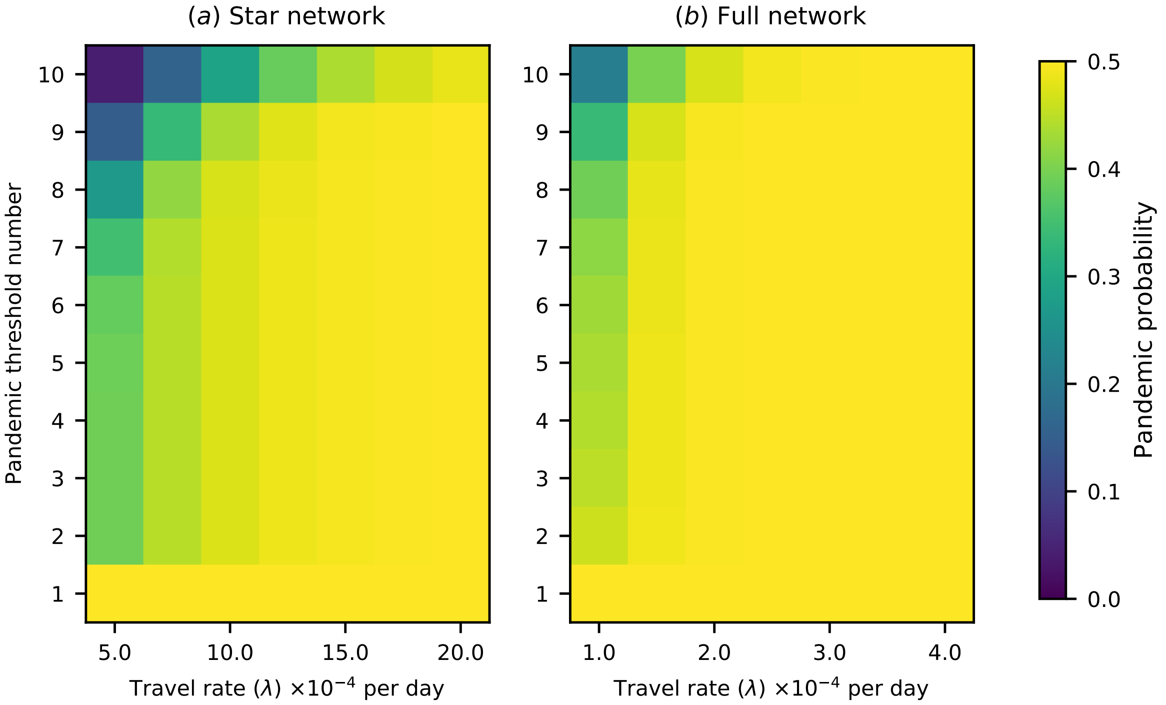

Figure S1: Analogous to Figure 2 in the main text, but with the travel rate for the star network increased to 5 times that for the full network, so as to keep the overall motility (i.e. total volume of travel) constant. We see that many features of Figure 2 are due to the difference in motility between the two networks. However, the difference in network structure between plot *(a)* and plot *(b)* is enough to make the plots distinct even at equal motility. In particular, we see that, while travel rate has a larger effect on pandemic probability in the star network than in the full network, the pandemic definition has only a slightly smaller influence on the pandemic probability in the full network at normal travel rates than in the star network at very high travel rates, which greatly increase the correlations across the network.

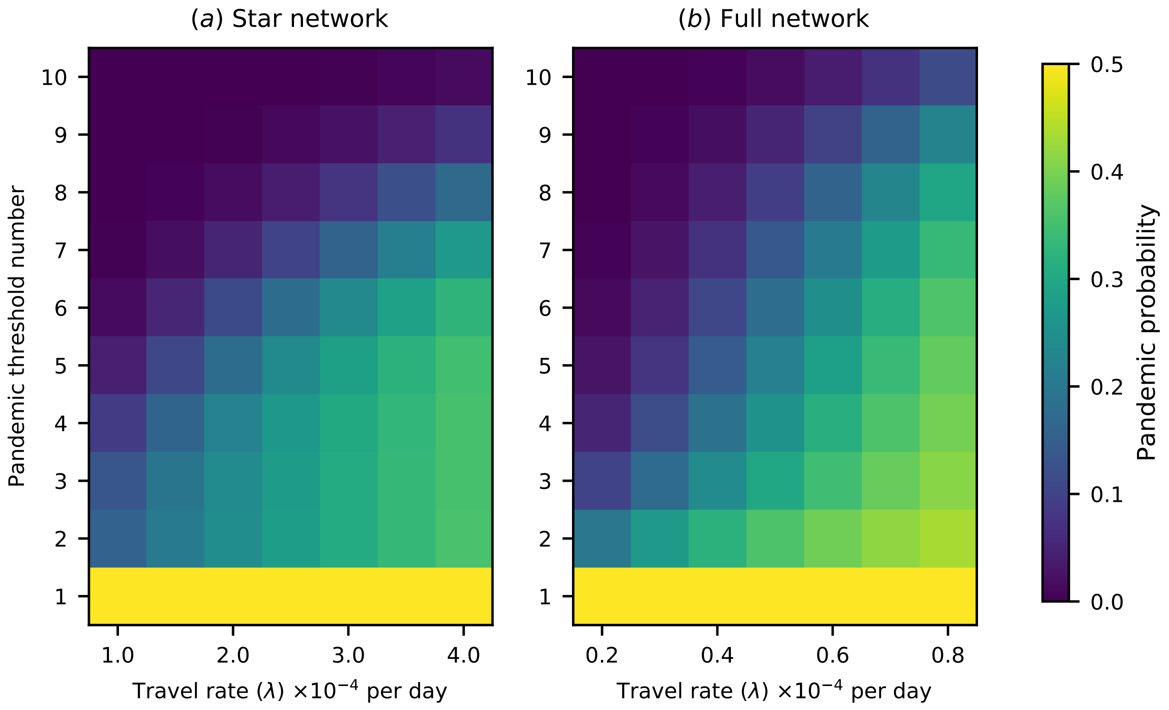

Figure S2: Analogous to Figure 2 in the main text, but with the travel rate for the full network decreased to 0.2 times that for the star network, so as to keep the overall motility constant. We see that many features of Figure 2 are due to the difference in motility between the two networks. However, the difference in network structure between plot *(a)* and plot *(b)* is enough to make the plots distinct even at equal motility. In particular, we see that pandemic definition has a larger effect in the full network at low travel rates than in the star network at normal travel rates.

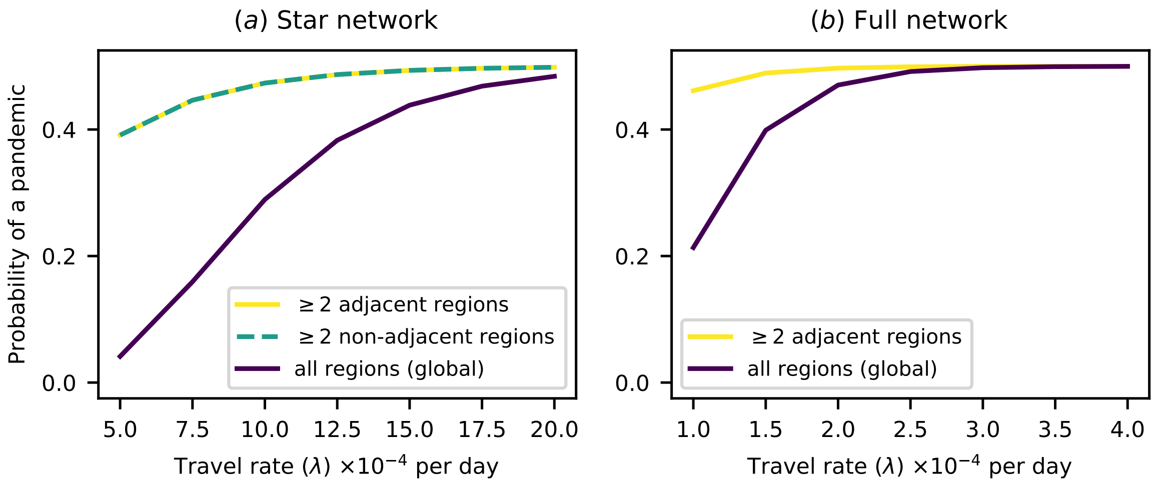

Figure S3: Analogous to Figure 3 in the main text, but with the travel rate for star network increased to 5 times that for the full network, so as to keep constant the overall motility. The very high travel rates mean that the difference between the pandemic probability for the transregional and interregional definitions becomes very small.

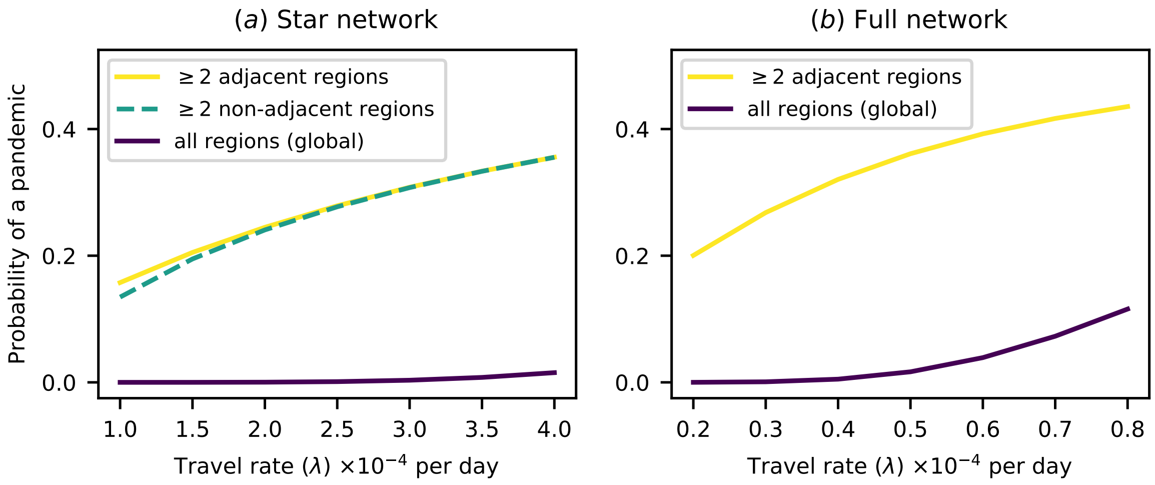

Figure S4: Analogous to Figure 3 in the main text, but with the travel rate for the full network decreased to 0.2 times that for the star network, so as to keep the overall motility constant. The very low travel rate greatly suppresses the probability of a global pandemic.

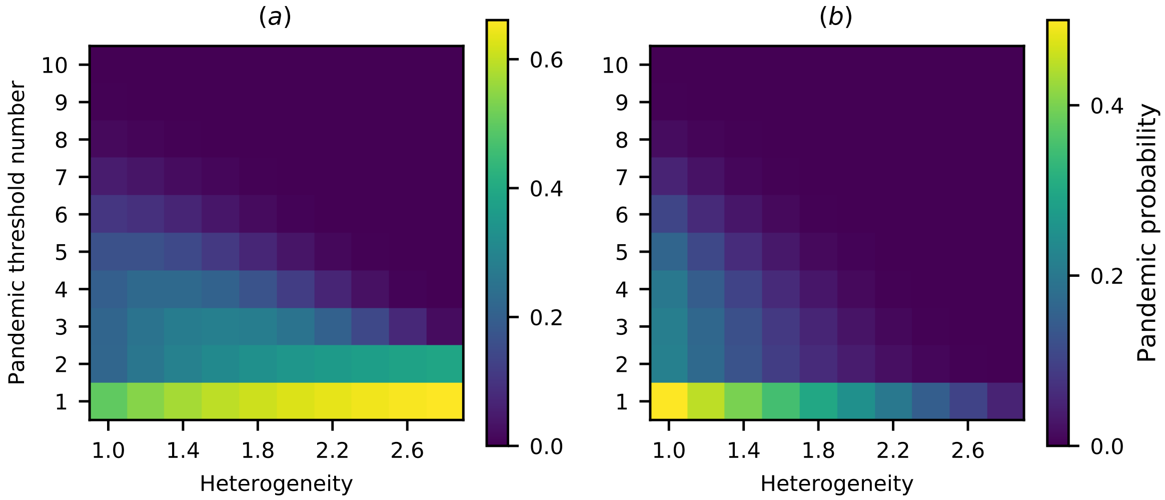

Figure S5: Analogous to Figure 6 in the main text, but made using a star network, with the outbreak seeded in a peripheral region and with the central region having the lower transmission rate. a) Pandemic probability for a pathogen emerging in a higher-transmission region. For low thresholds heterogeneity increases pandemic probability. For thresholds 3 and 4 the pandemic probability increases and then decreases as in Figure 6a in the main text. At thresholds higher than four the lowering transmission rate in the central population means that higher heterogeneity reduces pandemic probability. b) Pandemic probability for a pathogen emerging in a lower-transmission region. At all thresholds increasing heterogeneity decreases pandemic probability.

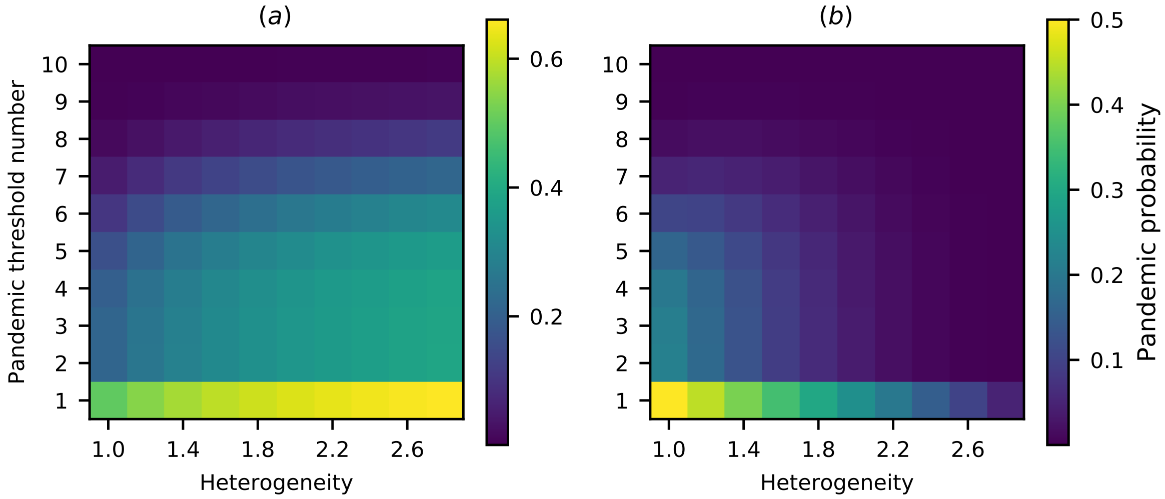

Figure S6: Analogous to Figure 6 in the main text, but made using a star network, with the outbreak seeded in a peripheral region and with the central region having the higher transmission rate. a) Pandemic probability for a pathogen emerging in a higher-transmission region. At all thresholds increasing heterogeneity increases pandemic probability. b) Pandemic probability for a pathogen emerging in a lower-transmission region. At all thresholds increasing heterogeneity decreases pandemic probability.

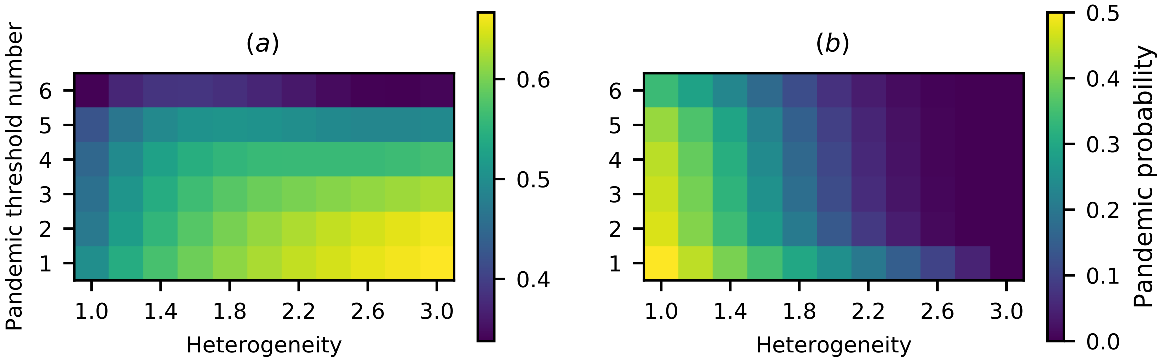

Figure S7: Analogous to Figure 6 in the main text, but made using a six-region network. a) Pandemic probability for a pathogen emerging in a higher-transmission region. For low thresholds heterogeneity increases pandemic probability, but at the 5 and 6-region thresholds pandemic probability grows and then decreases with increasing heterogeneity. b) Pandemic probability for a pathogen emerging in a lower-transmission region. At all thresholds increasing heterogeneity decreases pandemic probability.
